# Clinical implication of miRNA in acute myocardial infarction

**DOI:** 10.1002/jcla.23491

**Published:** 2020-08-13

**Authors:** Kosuke Miki, Teruhiko Imamura

**Affiliations:** ^1^ University of Toyama Toyama Japan; ^2^ Second Department of Internal Medicine University of Toyama Toyama Japan

**Keywords:** acute coronary syndrome, heart failure, hemodynamics, MACE

To the editor,

Early diagnosis and precise prognostic prediction are warranted in patients with acute myocardial infarction. Wang et al^1^ found miRNA as a novel diagnostic and prognostic biomarker in patients with acute myocardial infarction. Several concerns should improve their findings.

As the authors mentioned, longitudinal follow‐up of miRNA levels is warranted in addition to the cross‐sectional assessment. The levels of miRNA were measured at 6 hours following the admission, but it is unclear whether the measurements were conducted before or after coronary revascularization. Biomarkers that increase at earlier phase than the conventional ones would be clinically beneficial.

There are already many diagnostic biomarkers, including troponin and creatinine kinase, as well as many prognostic variables, including TIMI score and left ventricular ejection fraction.^2^ Further comparisons with these variables would improve their findings. For the prognostic comparison, a time‐to‐event analysis instead of the logistic regression analyses might be recommended.

Biomarkers are in general affected by comorbidities. For example, the plasma level of B‐type natriuretic peptide is elevated in patients with chronic kidney disease.^2^ The association between miRNA and such comorbidities would be helpful for clinicians to interpret the level of miRNA in the daily practice.

## Funding information

TI receives grant support from JSPS KALENHI: JP20K17143.
